# Case Report: An imported severe case of paediatric scrub typhus with Karp B subgenotype in non-endemic Northern China, Beijing

**DOI:** 10.3389/fped.2026.1733143

**Published:** 2026-04-17

**Authors:** Yiyang Mao, Qiuchi Lv, Siqi Chen, Lijuan Wang, Kechun Li, Zhengde Xie, Feifei Yin, Lili Xu, Quan Wang, Chengsong Zhao

**Affiliations:** 1 Department of Paediatric Critical Care Medicine, Beijing Children’s Hospital, National Center for Children’s Health, Capital Medical University, Beijing, China; 2 Beijing Key Laboratory of Core Technologies for the Prevention and Treatment of Emerging Infectious Diseases in Children, National Clinical Research Center for Respiratory Diseases, National Key Discipline of Pediatrics, Capital Medical University, Beijing, China; 3 Beijing Research Center for Respiratory Infectious Diseases, Beijing Pediatric Research Institute, Beijing Children’s Hospital, National Center for Children’s Health, Capital Medical University, Beijing, China; 4 Research Unit of Critical Infection in Children, Chinese Academy of Medical Sciences, Beijing, China; 5 Hainan Medical University-the University of Hong Kong Joint Laboratory of Tropical Infectious Diseases, Key Laboratory of Tropical Translational Medicine of Ministry of Education, School of Basic Medicine and Life Sciences, Hainan Medical University, Haikou, Hainan, China; 6 Department of Respiratory, Beijing Children’s Hospital, Beijing Research Center for Respiratory Infectious Diseases, National Clinical Research Center for Respiratory Diseases, National Center for Children’s Health, Capital Medical University, Beijing, China

**Keywords:** *Orientia tsutsugamushi*, paediatric case, scrub typhus, severe infection, 56-kDa type specific antigen gene

## Abstract

Scrub typhus, a zoonosis caused by *Orientia tsutsugamushi* (*O. tsutsugamushi*), remains a significant public health threat in the Asia-Pacific region. This disease is transmitted through the bite of infected trombiculid mite larvae (chiggers) and typically manifests as acute undifferentiated fever during the early stage. Despite the availability of targeted antibiotic therapies, delayed diagnosis frequently leads to severe complications and fatal outcomes. Here, we report a severe imported paediatric case in Beijing, a city in China's temperate zone, involving a 12-year-old girl with a recent travel history to Yunnan Province. The patient presented with fever, characteristic eschar, regional lymphadenopathy, and septic shock, ultimately progressing to multiorgan dysfunction syndrome. Whole-genome metagenomic next-generation sequencing (mNGS) of blood, cerebrospinal fluid (CSF), and sputum samples revealed *O. tsutsugamushi* with high sequence read counts, whereas blood cultures remained negative for other bacterial pathogens. Subsequent PCR amplification and Sanger sequencing confirmed the mNGS findings. Phylogenetic analysis of the TSA56 gene classified the strain within the Karp cluster. Serological analysis revealed the presence of *O. tsutsugamushi*-specific IgM and IgG antibodies. This severe paediatric case highlights the importance of considering travel-associated scrub typhus in the differential diagnosis of febrile illnesses in non-endemic regions. This is particularly relevant for patients with a history of insect bites in areas known to be endemic for *O. tsutsugamushi*.

## 1 Background

Scrub typhus, a natural-focal infectious disease caused by *Orientia tsutsugamushi*(*O. tsutsugamushi*), poses a significant public health concern in the Asia-Pacific region (1–8). In mainland China, cases are geographically clustered in tropical/subtropical provinces, including Hainan, Guangdong, Guangxi, and Yunnan, with the peak incidence occurring from June to July and from October to November (2). Scrub typhus is transmitted through the bite of infected trombiculid mite larvae (chiggers), which parasitize reservoir hosts such as rodents and insectivores. Human infection occurs following the bite of infected trombiculid mite larvae, with an incubation period of 6–21 days (5, 8, 9). The characteristic clinical hallmark is an eschar at the inoculation site, accompanied by regional lymphadenopathy and fever (6). Patients with severe disease may develop systemic manifestations, including pneumonitis, acute respiratory distress syndrome (ARDS), myocarditis, encephalitis, hepatitis, nephritis, disseminated intravascular coagulation (DIC), and haemophagocytic syndrome. In advanced stages, multiorgan dysfunction syndrome may occur, significantly increasing morbidity and mortality (10–14).

Here, we report a severe imported paediatric scrub typhus case diagnosed in Beijing, a non-endemic region of northern China. The pathogen was identified as *O. tsutsugamushi* Karp B subgenotype by metagenomic next-generation sequencing and confirmed by PCR and serology. This case highlights the importance of early recognition of travel-associated scrub typhus in non-endemic areas.

## 2 Case presentation

A previously healthy 12-year-old female was admitted to the emergency department on February 12, 2025, with an 8-day history of intermittent fever (peaking at 40 °C), a 7-day history of headache, and an 8-hour history of dizziness and cold extremities. The febrile illness began with low-grade fever that progressively worsened. Despite empirical intravenous cephalosporins, unidentified herbal remedies, and supportive fluids administered at a local clinic, her condition did not improve. Two days before admission (February 10, 2025), the patient experienced sudden-onset blurred vision upon standing at night, fell to the ground, and experienced convulsions that resolved on their own in approximately one minute. She was reportedly afebrile during the ictal phase, although no objective temperature measurement was performed. Epidemiological investigation revealed travel that she had travelled to Xishuangbanna, Yunnan Province—a recognized endemic focus for *O. tsutsugamushi,* two weeks before symptom onset. The patient had prolonged activity in wooded areas within a region known to be endemic for *O. tsutsugamushi*, where trombiculid mite larvae bites are the established mode of transmission.

**Figure 1:**
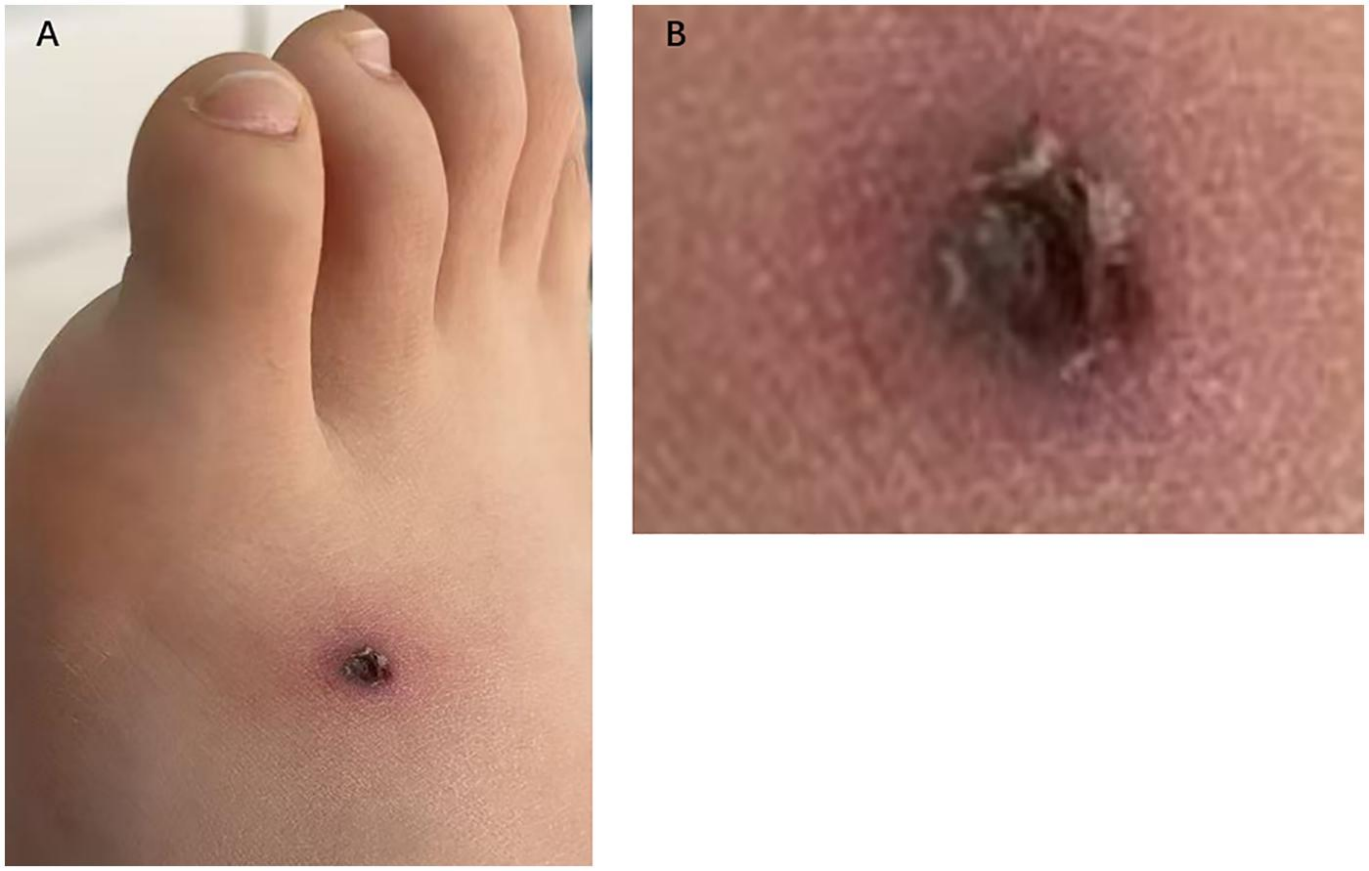
Characteristic eschar on the dorsum of the left foot. **(A)** Overall view of the left foot showing the dark black lesion with an approximate diameter of 10 mm. **(B)** Close-up view of the lesion, highlighting its characteristics.

Upon admission, the patient was hemodynamically unstable with the following vital signs: afebrile (36.9 °C), tachypneic (33 breaths/min), tachycardic (136 bpm), and hypotensive (77/32 mmHg). Hemodynamic stability was partially achieved with norepinephrine infusion (0.4 μg/kg/min), yielding a blood pressure of 113/63 mmHg. A 10-mm characteristic eschar was observed on the dorsum of the left foot (Figure 1). Multiple round purple petechiae were scattered along both sides of the spine. Cervical lymphadenopathy was noted, with bilateral firm, mobile nodes measuring 5 mm; no other peripheral lymphadenopathy was detected. Respiratory assessment while on nasal continuous positive airway pressure (NCPAP) demonstrated increased work of breathing, with tachypnea and suprasternal retractions. Auscultation revealed bilateral coarse crackles and diffuse moist rales. Abdominal examination revealed mild generalized guarding and tenderness, with positive rebound tenderness at both McBurney's point and the contralateral McBurney's point. The liver edge was palpated 2 cm below the costal margin and was soft in consistency, while the spleen was also palpable. No additional pathological findings were detected.

Laboratory evaluation revealed anaemia (haemoglobin 95 g/L, reference range: 118–156 g/L) and thrombocytopaenia (platelet count 51 × 10^⁹^/L, reference range: 150–407 × 10^⁹^/L). Furthermore, markedly elevated inflammatory markers were noted, including procalcitonin (2.89 ng/mL, reference range: ≤0.5 ng/mL) and C-reactive protein (97 mg/L, reference range: <10 mg/L). Hepatic dysfunction was evidenced by elevated transaminases: aspartate aminotransferase (AST) 139.3 U/L, (reference range: 14–44 U/L) and alanine aminotransferase (ALT) 74.8 U/L (reference range: 7–30 U/L). The detailed laboratory findings are summarized in Table 1.

**Table 1:** Laboratory findings on admission and at recovery (hospital Day 16).

Parameter	On admission (February 12, 2025)	Hospital day 16 (February 28, 2025)	Reference range (pediatric)^a^
White blood cells (×10^9^/L)	4.99	4.35	4.3–11.3
Red blood cells (×10^12^/L)	3.37	3.28	4.2–5.7
Hemoglobin (g/L)	95	98	118–156
Platelets (×10^9^/L)	51	157	150–407
Hematocrit (%)	26.3	27.8	36–46
Neutrophils (×10^9^/L)	4.09	0.95	1.6–7.8
Lymphocytes (×10^9^/L)	0.72	3	1.5–4.6
C-reactive protein (mg/L)	97	<10	<10
Procalcitonin (ng/mL)	2.89	-	≤0.5
Albumin (g/L)	26.5	-	39–54
N-terminal pro-B-type natriuretic peptide (pg/mL)	1,780	-	<450
B-type natriuretic peptide (pg/mL)	-	<10	0–100
Alkaline phosphatase (U/L)	74	-	81–454
Aspartate aminotransferase (U/L)	139.3	33.4	14–44
Alanine aminotransferase (U/L)	74.8	42.8	7–30
Ferritin (ng/mL)	6,605	432.1	6–159
Amylase (U/L)	55	135	0–125
Lipase (U/L)	46.7	169.1	0–39
Pancreatic amylase (U/L)	32	92	17–115
Prothrombin time (s)	16.3	-	9.4–12.5
Fibrinogen (g/L)	1.35	-	2–4
Activated partial thromboplastin time (s)	49.5	-	25.1–38.4
Thrombin time (s)	18.9	-	14.3–18.8
D-dimer (mg/L)	14.64	-	0–0.243

a Reference ranges are influenced by multiple factors, including the patient population and the laboratory methods used. The ranges provided are those used at Beijing Children's Hospital for pediatric patients and may not be applicable to all populations. A dash (—) indicates that the test was not performed on that day.

**Figure 2:**
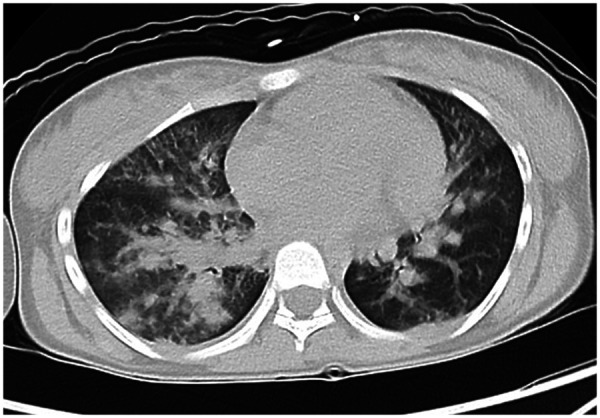
Chest CT showing bilateral multifocal pulmonary abnormalities, including patchy and nodular opacities, linear densities, and ground-glass opacities. Accompanying findings included thickening of the interlobular septa and interlobar pleura, as well as bilateral lower lobe pleural thickening.

Imaging studies revealed pulmonary involvement. Chest CT showed bilateral multifocal parenchymal abnormalities, including patchy consolidations, nodular lesions, linear opacities, and ground-glass opacities. These findings were accompanied by interlobular septal thickening and bilateral lower lobe pleural thickening (Figure 2). Abdominal CT revealed splenomegaly, and abdominal ultrasound revealed pelvic ascites with a maximum depth of approximately 2.2 cm. No significant abnormalities were detected on brain CT. Cerebrospinal fluid (CSF) analysis revealed clear fluid, with negative bacterial culture and normal biochemical parameters. The patient was admitted to the paediatric intensive care unit (PICU) for management of septic shock.

Following admission, the patient received standard supportive care, including NCPAP and subsequent endotracheal intubation with positive pressure ventilation due to hypoxemia, pulmonary edema and shock. Continuous infusions of midazolam and fentanyl were administered to maintain sedation and analgesia for mechanical ventilation. Following intubation, copious, lightly blood-tinged secretions were noted. Concurrent management included norepinephrine infusion and 50 mg hydrocortisone for blood pressure support, along with fresh frozen plasma transfusion to address coagulopathy. In view of the diagnosis of septic shock, empirical antibiotic therapy with meropenem and vancomycin was initiated. Adjunctive immunomodulatory therapy included 20 g intravenous immunoglobulin (IVIG) and 240 mg tocilizumab for cytokine storm.

On hospital Day 2 (February 13, 2025), mNGS was performed on blood, CSF, and sputum samples to identify the causative pathogen. DNA of *O. tsutsugamushi* was detected in all three specimens. Also, bronchoalveolar lavage was performed to obtain lower respiratory tract specimens (bronchoalveolar lavage fluid, BALF) for molecular detection and genotyping. The diagnosis of severe scrub typhus was subsequently confirmed by the convergence of clinical, epidemiological, and molecular evidence. The patient's recent travel to an endemic region of Yunnan Province, where exposure to infected trombiculid mite larvae is a known risk factor, provided a strong epidemiological link. This was further supported by characteristic clinical findings, including fever, regional lymphadenopathy, and an eschar on the left foot. Definitive confirmation was achieved through molecular detection of *O. tsutsugamushi* in multiple specimen types. In light of these findings, antimicrobial therapy was promptly switched to intravenous doxycycline (2.2 mg/kg every 12 h). On hospital Day 5 (February 16, 2025), laboratory testing revealed an elevated serum lipase level of 174 U/L (reference range: 0–39 U/L), prompting the initiation of somatostatin to suppress pancreatic secretion. By hospital Day 7 (February 18, 2025)—six days after initiating doxycycline—the patient exhibited marked clinical improvement, accompanied by normalization of inflammatory markers, including C-reactive protein (CRP). Complete neurological recovery was achieved by hospital Day 9 (February 20, 2025), meeting the extubation criteria, followed by successful discharge from the PICU on hospital Day 11 (February 22, 2025). The patient achieved full recovery during subsequent follow-up visits.

## 3 Materials and methods

### 3.1 Whole-genome metagenomic next-generation sequencing

Blood, CSF, and sputum samples collected on hospital Day 2 were subjected to whole-genome mNGS analysis. The samples were aseptically collected in sterile containers and transported to Hugobiotech Co., Ltd. (Beijing, China). DNA was extracted from blood samples using a QIAamp DNA Micro Kit (QIAGEN, Hilden, Germany), and DNA libraries were constructed with the QIAseq™ Ultralow Input Library Kit for Illumina (QIAGEN, Hilden, Germany) according to the manufacturer's instructions. A Qubit fluorometer (Thermo Fisher Scientific, MA, USA) and an Agilent 2100 Bioanalyzer (Agilent Technologies, Palo Alto, CA, USA) were utilized to assess the quality and quantity of each library. Only libraries that met the quality standards were sequenced on the NextSeq 550 platform (Illumina, San Diego, CA, USA), which targeted approximately 20 million 75-bp single-end reads per library. The expected number of sequencing reads was 20 million reads per sample. The actual yields were 22.2 million reads for the sputum sample, 25.5 million for the CSF sample, and 16.8M reads for the blood sample.

After obtaining the raw data, bcl2fastq (v2.20.0.422) was employed to convert the BCL files into FASTQ format and to demultiplex the data for each sample. Next, fastp (v0.24.0) was run with default parameters to remove adapter sequences, low-quality reads, and sequences with a high proportion of ambiguous bases (“N”), thereby generating high-quality reads with a minimum length of 50 bp (15). Subsequently, BWA (0.7.15) in “mem” mode was used to align the reads to the human reference genome (GRCh38.101), thereby removing host-derived sequences (16). The remaining reads were then aligned using BWA to an in-house microbial genome database compiled from the NCBI RefSeq and GenBank databases. Microbial identification was then performed based on the alignment results.

Negative controls (sterile deionized water) and positive controls (known quantities of synthetic fragments) were included in each experimental batch and processed using the same wet-laboratory and bioinformatics procedures. A bacterial result was considered positive if either of the following criteria were met: (1) the coverage ranked among the top 10 for the corresponding species/genus and the organism was absent in the NTC; or (2) the reads per million (RPM) ratio between the sample and NTC (RPM_ample_/RPM_NTC_) exceeded 10, provided that the RPM_NTC_ was not zero.

The raw sequence data generated in this study have been deposited in the Genome Sequence Archive (Genomics, Proteomics & Bioinformatics 2021) in National Genomics Data Center (Nucleic Acids Res 2022), China National Center for Bioinformation/Beijing Institute of Genomics, and are publicly accessible at https://ngdc.cncb.ac.cn under BioProject accession number PRJCA039866.

### 3.2 Serological testing

Serum sample from patients in the acute phase were analyzed for *O. tsutsugamushi*-specific IgM and IgG antibodies using a colloidal gold-based immunochromatographic assay (Wantai BioPharm, Beijing, China), following the manufacturer's protocol. Infection with scrub typhus typically allows for the detection of specific IgM and IgG antibodies within the first week of illness, with the highest positivity rates observed during the second week.

### 3.3 Molecular detection and genotyping

DNA was extracted from sputum and BALF samples using an automated nucleic acid extraction system (Shanghai Zhongyuan Huiji Biotech, China) according to the manufacturer's protocol, which is optimized for complex respiratory matrices. Two PCR approaches were used in this study: a commercial real-time quantitative PCR kit for screening detection, and an in-house nested PCR targeting the TSA56 gene for genotyping.

1. Real-time PCR: Screening was performed using the Zhongyuan Huiji (Zybio) *O. tsutsugamushi* real-time PCR detection kit. The kit includes specific primers and probes targeting the 47-kDa outer membrane protein-encoding gene (species-specific marker), the GroEL heat shock protein-encoding gene (highly conserved region), and the 56-kDa type-specific antigen gene (TSA56) (genotyping marker). The primer and probe sequences are proprietary and not publicly disclosed by the manufacturer; therefore, we are unable to provide the exact sequences in this manuscript. Fluorescence signals were monitored in independent reaction systems for qualitative detection of *O. tsutsugamushi* genomic targets. Positive and negative controls, both provided with the kit, were included in each run according to the manufacturer's instructions. A sample was considered positive if amplification curves for at least two molecular targets demonstrated exponential growth with cycle threshold (Ct) values below 40.
2. Nested PCR: To improve the specificity and sensitivity of *O. tsutsugamushi* genotyping, the 56-kDa type-specific antigen gene (TSA56) was amplified using nested PCR, following previously established protocols from our group (17, 18). The expected amplicon size of was 483 bp. The first-round PCR used the forward primer: 5′-TCAAGCTTATTGCTAGTGCAATGTCTGC-3′ and reverse primer 5′-AGGGATCCCTGCTGCTGTGCTTGCTGCG-3′. The second-round PCR used the forward primer 5′-GATCAAGCTTCCTCAGCCTACTATAATGCC-3′ and reverse primer 5′-CTAGGGATCCCGACAGATGCACTATTAGGC-3′. The amplified products were purified and subjected to bidirectional Sanger sequencing.

A 487-bp partial fragment of the *O. tsutsugamushi* TSA56 gene was obtained and designated BJ-HYM. The nucleotide sequence has been deposited in the GenBank database under accession number PV395367.1.

### 3.4 Phylogenetic analysis

To characterize the genetic variations of the *O. tsutsugamushi* TSA56 gene fragment obtained in this study, reference TSA56 gene sequences from various geographical regions were retrieved from the GenBank database. Nucleotide sequence alignment was performed using MAFFT version 5 (19). Following alignment, the sequences were trimmed using BioEdit software. Genetic characterization and sequence homology analysis were performed using DNASTAR MegAlign software. Multiple sequence alignment was performed using ClustalW software with default parameters. Phylogenetic analysis was conducted using MEGA-X software under the maximum likelihood (ML) framework, with the optimal substitution model selected based on the Bayesian information criterion (BIC) (20). Bootstrap resampling with 1,000 replicates was performed. This analysis provided robust support for the tree topology, enhancing the reliability of the inferred phylogenetic relationships.

## 4 Results

### 4.1 Whole-genome mNGS findings

*O. tsutsugamushi* DNA was detected by mNGS in blood (581 reads), CSF (431 reads), and sputum (932 reads). In addition, mNGS of sputum also revealed DNA sequences from several opportunistic pathogens, including *Prevotella histicola* (7 reads), *Veillonella parvula* (6 reads), *Prevotella loescheii* (4 reads), *Prevotella oris* (3 reads), *Trueperella pyogenes* (3 reads), *Alloscardovia* (2 reads), and *Megasphaera micronuciformis* (1 reads). Due to their low read counts and relative abundance, together with negative results from conventional bacterial cultures, these bacterial species were not considered to be causative pathogens.

### 4.2 Serological findings

The patient developed fever on February 4, 2025, 8 days prior to admission, and a serum sample was collected for serological testing on February 19, 2025. Serological testing showed weak positivity for IgM antibodies and strong positivity for IgG antibodies. The serological diagnosis of scrub typhus is characterized by specific antibody kinetics, with IgM and IgG antibodies typically becoming detectable within 7 days after symptom onset. Peak seropositivity rates for both immunoglobulins are reached during the second week of infection. Although these antibody tests provide valuable supportive evidence for the diagnosis of acute scrub typhus, the results should be interpreted in conjunction with clinical findings and other diagnostic modalities.

### 4.3 Phylogenetic analysis

**Figure 3:**
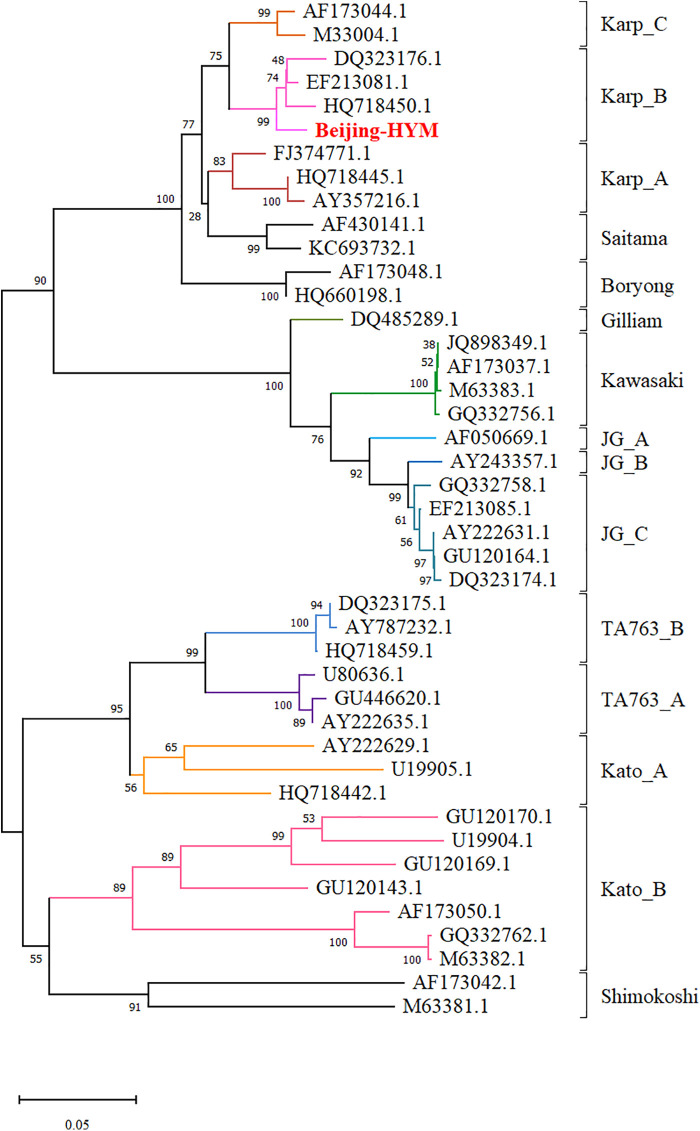
Phylogenetic analysis based on TSA56 gene sequences, showing the classification of *Orientia tsutsugamushi* strains into genotypes and genogroups. Bootstrap support values are indicated at the corresponding nodes. The strain identified in this study (BJ-HYM) is highlighted in red.

Phylogenetic analysis was performed based on a 487-bp fragment of the TSA56 gene to determine the genotype of the *O. tsutsugamushi* strain identified in this case. The obtained sequence, designated “BJ-HYM” and deposited in GenBank under accession number PV395367.1, was aligned with reference sequences representing all major *O. tsutsugamushi* genotypes retrieved from GenBank. Phylogenetic reconstruction using the maximum likelihood method in MEGA X with 1,000 bootstrap replicates placed the BJ-HYM strain within the Karp B subgenotype clade with high bootstrap support. Nucleotide homology comparisons revealed that BJ-HYM shared the closest phylogenetic relationship with strains DQ323176.1 (Taiwan, China, 2007), EF213081.1 (Thailand, 2008), and HQ718450.1 (Vietnam, 2014). Among these, EF213081.1 (Thailand, 2008) exhibited the highest sequence similarity, with 94.6% nucleotide identity to BJ-HYM. The strain was phylogenetically distinct from other Karp subgenotypes (Karp A and Karp C) as well as from other major genotypes, including Gilliam, Kato, Kawasaki, Boryong, Saitama, Shimokoshi, TA763, and JG (Figure 3). This genotyping result is consistent with the epidemiological pattern observed in southwestern China (Yunnan Province) and represents the first documented detection of the Karp B genotype in Beijing.

## 5 Discussion and conclusions

Scrub typhus, caused by *O. tsutsugamushi*, primarily affects field-working farmers through vector exposure in endemic regions. Compounded by limited protective knowledge and generally lower immunity, this population faces heightened infection risks (2, 7, 10, 12). Notably, epidemiological surveillance has reveals a distinct prevalence pattern in Southwest China (Yunnan and Sichuan provinces), where children under 5 years of age exhibit the highest disease prevalence (1). This epidemiological pattern may be associated with intensive agricultural activities in mountainous areas.

Scrub typhus typically presents as acute undifferentiated fever in the early stage, which often leads to diagnostic uncertainty. The incubation period typically ranges from 6 to 21 days after exposure (5, 8, 9). The diagnosis of scrub typhus typically relies on a combination of epidemiological history, clinical manifestations, and serological tests such as ELISA (3, 10, 12, 13). A characteristic clinical feature is the eschar, which forms at the site of the bite of infected trombiculid mite larvae, typically in areas where skin surfaces appose, such as the axilla, groin, and inguinal regions (8). The distribution of eschars differs significantly between males and females. In females, eschars are mainly found on the chest and abdomen, whereas in males, they are predominantly located in the axilla, groin, and genital areas (9). Severe infection can lead to systemic complications, including pneumonitis, ARDS, myocarditis, encephalitis, hepatitis, nephritis, DIC, and haemophagocytic syndrome. Advanced disease may progress to multiorgan dysfunction, resulting in high morbidity and mortality (6). It is noteworthy that scrub typhus has emerged as a cause of acute encephalitis syndrome **(**AES) among children in some countries (21, 22).

Despite the presence of these diagnostic clues, delays in diagnosis remain common. Current surveillance data indicate a median diagnostic delay of 6–8 days for scrub typhus in mainland China (5, 9, 14). Although targeted antimicrobial therapy (e.g., doxycycline, azithromycin) has demonstrated clinical efficacy, disease progression to severe complications and fatal outcomes frequently occurs due to delayed treatment initiation (2, 9, 10, 12, 13, 23). Mortality rates exceed 6% in untreated cases, with paediatric populations (<10 years) exhibiting a median case fatality rate of 1.8% (1, 24).

In this case, the patient experienced a 9 day delay from symptom onset to definitive diagnosis after hospitalization. Physical examination revealed significant findings, including an eschar and lymphadenopathy. However, in this case, the eschar was located in a non-typical site. Although a typical eschar was present, the delayed diagnosis may be attributed to limited awareness of scrub typhus among parents and physicians in the non-endemic region. By the time of diagnosis, the disease had progression had progressed to systemic involvement, including myocarditis (manifesting as shock and arrhythmia), pneumonitis/ARDS, encephalitis, and nephritis.

The diagnosis was confirmed by whole-genome mNGS of blood, CSF and sputum samples, all of which tested positive for *O. tsutsugamushi*, whereas blood cultures remained negative for bacterial pathogens. PCR offers the advantages of high sensitivity, strong specificity, and a short turnaround time, making it widely used in molecular biology and other fields (25). Nested PCR and real-time PCR are both commonly used for the diagnosis of scrub typhus. In addition to the TSA56 gene, the 16S rRNA gene and the 47-kDa high-temperature requirement A gene can also be used for the detection of *O. tsutsugamushi* (26). Common sample types for PCR detection of scrub typhus include blood, eschar swabs, CSF, sputum, and BALF. Blood is the most frequently used sample type. For patients with a typical eschar, swabbing the surface of the lesion often yields a good positive rate. For patients with complications such as pneumonia or meningitis, sputum, BALF, or CSF may be collected for testing (27). Of note, detection of *O. tsutsugamushi* DNA in sputum is uncommon. This may be attributable to the presence of blood in the sputum, which could have served as the source of *O. tsutsugamushi* DNA. Subsequent PCR and Sanger sequencing confirmed the mNGS findings. In addition, serological testing demonstrated the presence of *O. tsutsugamushi*-specific IgM and IgG antibodies. The patient developed fever on February 4, 2025, 8 days before admission. A serum sample was collected and subjected to serological testing on February 19, 2025. The interval from disease onset to serological testing was 16 days, which is consistent with the previously mentioned trend that the positive rates of *O. tsutsugamushi*-specific IgM and IgG antibodies peak in the second week after disease onset. However, since only one serum sample was tested, the possibility that the patient's anti-*O. tsutsugamushi* antibodies resulted from prior exposure cannot be excluded. Therefore, serological testing usually serves as a clinical auxiliary diagnostic method for scrub typhus but cannot be used as the sole diagnostic criterion.

In this case, the patient was initiated on empirical broad-spectrum antibiotics (meropenem and vancomycin) upon admission for septic shock, followed by targeted therapy with intravenous doxycycline after confirmation of *O. tsutsugamushi* infection. Supportive treatments included anti-inflammatory therapy with IVIG and tocilizumab, vasopressor support with norepinephrine, mechanical ventilation for acute respiratory failure, and somatostatin to suppress pancreatic secretion. Doxycycline is widely regarded as the drug of choice for the treatment of scrub typhus. It demonstrates excellent efficacy and is widely used in clinical practice (28).

The TSA56 gene, which encodes a major outer membrane protein with high antigenic diversity, serves as the primary molecular target for genotyping *O. tsutsugamushi* (5, 12, 14, 29, 30). The current consensus recognizes Gilliam, Karp, and Kato as the predominant prototypes strains globally (31–34). Molecular epidemiological studies in China have identified multiple circulating genotypes of *O. tsutsugamushi,* including Gilliam, Kato, Karp, Kuroki, Shimokoshi, TA763, and Kawasaki (3). The Karp genotype is predominant worldwide, accounting for approximately 50% of all infections (5, 33). Further surveillance has revealed distinct geographical heterogeneity in the distribution of *O. tsutsugamushi* genotypes across China. The Kawasaki genotype predominates in Shandong Province in northern China (30), whereas southern regions, particularly Guangdong and Taiwan, exhibit dominance of the Karp genotype (3, 6). Guangxi exhibits co-circulation of the Gilliam and Karp genotypes (35). Yunnan Province displays predominant Karp and Kato genotypes, which together account for approximately 50% of the scrub typhus cases in China (3, 8, 34). Sequencing of the TSA56 gene and multilocus sequence typing (MLST) have revealed the presence of second-generation subgenotypes within the Karp genotype, designated Karp A, Karp B, and Karp C. These subgenotypes also exhibit a broad geographical distribution. The Karp A subgenotype has been detected in Hainan Province (China), Taiwan, Japan, South Korea, Thailand, and Papua New Guinea. The Karp B subgenotype has been found in Hainan Province (China), Vietnam, Taiwan, and Thailand. The Karp C subgenotype has an even wider distribution, having been identified in Hainan, Zhejiang, and Fujian provinces of China, as well as in Taiwan, Cambodia, Thailand, Vietnam, and Malaysia (32). Phylogenetic analysis revealed that the strain identified in this case belonged to the Karp B subgenotype, consistent with the predominant genotype pattern observed in Yunnan Province. This case also represents the first documented detection of the Karp B subgenotype in Beijing.

This report describes a case of severe travel-associated scrub typhus occurring in a non-endemic region of northern China during winter. Although the patient presented with characteristic eschar and regional lymphadenopathy, her travel history to a region known to be endemic for *O. tsutsugamushi*—where trombiculid mite larvae bites are the established vector—was critical for diagnostic confirmation. Scrub typhus has been documented in all Chinese provinces except Shanghai (13, 36), with infection risks extending beyond traditional high-risk occupational groups. These epidemiological patterns underscore the need for heightened clinical awareness. We recommend: (1) systematic evaluation of exposure history in patients presenting with unexplained fever to facilitate timely diagnosis; (2) targeted preventive measures for individuals in endemic areas, including avoidance of direct contact with vegetation and use of protective clothing; and (3) prompt medical evaluation upon development of characteristic signs, such as persistent fever, eschar formation, or regional lymphadenopathy. Effective risk communication regarding potential exposures remains essential for early disease recognition and management.

## Data Availability

The original contributions presented in the study are publicly available. This data can be found here: https://www.ncbi.nlm.nih.gov/nuccore/PV395367, accession number: PV395367.
